# A phase I oncolytic virus trial with vesicular stomatitis virus expressing human interferon beta and tyrosinase related protein 1 administered intratumorally and intravenously in uveal melanoma: safety, efficacy, and T cell responses

**DOI:** 10.3389/fimmu.2023.1279387

**Published:** 2023-10-31

**Authors:** Katherine E. R. Smith, Kah-Whye Peng, Jose S. Pulido, Adam J. Weisbrod, Carrie A. Strand, Jacob B. Allred, Alysha N. Newsom, Lianwen Zhang, Nandakumar Packiriswamy, Timothy Kottke, Jason M. Tonne, Madelyn Moore, Heather N. Montane, Lisa A. Kottschade, Robert R. McWilliams, Arkadiusz Z. Dudek, Yiyi Yan, Anastasios Dimou, Svetomir N. Markovic, Mark J. Federspiel, Richard G. Vile, Roxana S. Dronca, Matthew S. Block

**Affiliations:** ^1^ Department of Medical Oncology, Mayo Clinic, Rochester, MN, United States; ^2^ Department of Molecular Medicine, Mayo Clinic, Rochester, MN, United States; ^3^ Department of Ophthalmology, Wills Eye Hospital, Philadelphia, PA, United States; ^4^ Department of Radiology, Mayo Clinic, Rochester, MN, United States; ^5^ Department of Biostatistics and Informatics, Mayo Clinic, Rochester, MN, United States; ^6^ Department of Hematology and Oncology, Mayo Clinic Florida, Jacksonville, FL, United States

**Keywords:** oncolytic virus, uveal melanoma, phase 1, immunotherapy, epitope spreading

## Abstract

**Introduction:**

Metastatic uveal melanoma (MUM) has a poor prognosis and treatment options are limited. These patients do not typically experience durable responses to immune checkpoint inhibitors (ICIs). Oncolytic viruses (OV) represent a novel approach to immunotherapy for patients with MUM.

**Methods:**

We developed an OV with a Vesicular Stomatitis Virus (VSV) vector modified to express interferon-beta (IFN-β) and Tyrosinase Related Protein 1 (TYRP1) (VSV-IFNβ-TYRP1), and conducted a Phase 1 clinical trial with a 3 + 3 design in patients with MUM. VSV-IFNβ-TYRP1 was injected into a liver metastasis, then administered on the same day as a single intravenous (IV) infusion. The primary objective was safety. Efficacy was a secondary objective.

**Results:**

12 patients with previously treated MUM were enrolled. Median follow up was 19.1 months. 4 dose levels (DLs) were evaluated. One patient at DL4 experienced dose limiting toxicities (DLTs), including decreased platelet count (grade 3), increased aspartate aminotransferase (AST), and cytokine release syndrome (CRS). 4 patients had stable disease (SD) and 8 patients had progressive disease (PD). Interferon gamma (IFNγ) ELIspot data showed that more patients developed a T cell response to virus encoded TYRP1 at higher DLs, and a subset of patients also had a response to other melanoma antigens, including gp100, suggesting epitope spreading. 3 of the patients who responded to additional melanoma antigens were next treated with ICIs, and 2 of these patients experienced durable responses.

**Discussion:**

Our study found that VSV-IFNβ -TYRP1 can be safely administered via intratumoral (IT) and IV routes in a previously treated population of patients with MUM. Although there were no clear objective radiographic responses to VSV-IFNβ-TYRP1, dose-dependent immunogenicity to TYRP1 and other melanoma antigens was seen.

## 1 Introduction

The treatment landscape for melanoma rapidly evolved after the introduction of immune checkpoint inhibitors (ICIs). Prior to ICI approval, the 5-year survival for melanoma was 18.3% in 2010, which has increased to 31.9% based on 2018 data (1). However, this gain in survival is largely due to advances in metastatic cutaneous melanoma (CM) (2–4). Uveal melanoma (UM) is a rare entity, with an incidence of 4.6 per million, and prognosis is poor with 2-year overall survival (OS) rates around 8% for metastatic UM (MUM) (5, 6). Unlike CM, MUM is less responsive to ICIs with a median OS of 11.3 months for ipilimumab/nivolumab compared to 72.1 months for CM, and a median OS of 15.7 months for pembrolizumab compared to 32.7 months in CM (7–9).

The difference in response to ICIs may in part be due to UM having a lower tumor mutational burden (TMB) and PD-L1 expression compared to CM, even when samples are analyzed from the same metastatic site (10). This leads to lower neoantigen expression and thus decreased recognition by tumor-specific T-cells (10–12). The most recent innovation for MUM is tebentafusp, an immune-mobilizing monoclonal T-cell receptor (TCR) against cancer (ImmTAC) targeting HLA-A*0201•gp100_280-288_ and CD3. This approach addresses the paucity of endogenous T cell recognition of UM and is associated with a median OS of 15.3 months (13). Despite this advancement, the majority of patients with MUM are HLA-A*0201 negative, thus due not qualify for tebentafusp. Overall, patients need access to more effective therapies.

Oncologic viruses (OV) are a novel approach to immunotherapy in that they can induce tumor death through both direct oncolysis and immune-mediated destruction. Currently, the only FDA-approved OV is talimogene laherparepvec (T-VEC), an intralesional injection of a modified herpes virus that leads to tumor lysis and granulocyte macrophage colony-stimulating factor (GM-CSF) delivery. T-VEC is used in unresectable CM based on improved response rates (RR) compared to GM-CSF alone (14). Additional OVs are under investigation in melanoma and other solid tumors with various viral vectors, transgenes, and routes of administration. The most common viral vectors studied have been adenovirus and herpes-simplex viruses (HSV), often with a GM-CSF transgene due to its ability to recruit dendritic cells (DCs) and natural killer cells (NKs), and the OV is usually given intratumorally (IT). There are few studies investigating intravenous (IV) administration with no studies prior to our trial evaluating concurrent IT and IV administration (15, 16).

Our study investigates the use of a novel OV, a vesicular stomatitis virus (VSV) vector modified to express interferon-beta (IFN-β) and Tyrosinase Related Protein 1 (TYRP1) (VSV-IFNβ-TYRP1). VSV as a vector is appealing since this virus does not naturally infect humans. In rare circumstances when humans are infected, they are asymptomatic or have a mild flu-like illness that resolves spontaneously within a few days and is not contagious to other humans (15, 16). Additionally, because humans are not typically infected with VSV, most patients do not have pre-existing neutralizing anti-VSV antibodies. VSV encoding IFN-β leads to rapid apoptosis of transformed cells while in normal cells the cytopathic effect is diminished due to the production of recombinant IFN-β (17). Additionally, IFN-β can inhibit cell growth, induce apoptosis, inhibit angiogenesis, and has immunomodulatory effects such as increasing T-cell responses via DCs leading to enhanced anti-tumor immune responses (18). We hypothesized that incorporation of TYRP1, a melanocyte differentiation antigen expressed in melanocytes and melanoma present in 65% of UM, may additionally upregulate an immune response to pigmented cells, thus improving immunogenicity (19). IV administration has previously been shown to be safe in a phase 1 trial of VSV-IFNβ with a sodium iodide symporter (VSV-IFNβ-NIS) in relapsed hematological malignancies, so we sought to evaluate the safety of administering our therapy IV in addition to IT (16). IT administration ensures virus delivery to the tumor to initiate an infection and prime the immune cells while IV administration delivers VSV-IFNβ-TYRP1 to other metastatic lesions.

We report the results of our phase 1 clinical trial investigating VSV-IFNβ-TYRP1 in previously treated patients with MUM, via IT and IV administration. The primary endpoint was safety. Efficacy was a secondary endpoint. Correlative studies focused on understanding viral pharmacokinetics (PK) and the immunological responses induced by VSV-IFNβ-TYRP1 therapy.

## 2 Materials and methods

The study was a phase 1 clinical trial offered at Mayo Clinic in Rochester, MN. The primary objective was to determine the safety profile of VSV-IFNβ-TYRP1 administered via IV and IT routes. Secondary objectives were to gather preliminary data on tumor response and progression-free survival (PFS). We investigated both viral and immunological correlates.

### 2.1 Production and preclinical rationale

Clinical-grade VSV-hIFNb-TYRP1 was produced by the Mayo Clinic Viral Vector Production Laboratory (VVPL) (https://www.mayo.edu/research/centers-programs/cancer-research/shared-resources-core-facilities-services/gene-virus-therapy/viral-vector-production-laboratory) in accordance with Good Manufacturing Practice. The virus was produced using a LaSt 293 HEK suspension cell line generated by the Mayo Clinic VVPL by adapting a GMP HEK293 adherent cell line to serum-free growth conditions. The virus was prepared in storage buffer (5% sucrose, 50 mM Tris-HCL [pH 7.4], 2 mM MgCl2), dispensed in cryotubes and stored at £ -65°C. Virus titer was determined by 50% tissue culture infective dose (TCID_50_) assay on BHK-21 cells. The titer of the preparation was 6.4 x 10^10^ TCID_50_ units/mL. Stability testing was performed in advance to clinical use to ensure post thaw stability during formulation at the pharmacy and in the delivery device under conditions of administration to the patient. Virus titer was routinely monitored throughout the duration of the clinical trial to ensure stability of virus titer during storage (full details available on request).

A prior study (NCT01628640) of VSV-IFNβ (lacking the TYRP1 antigen gene) resulted in a patient death after IT injection, which is thought due to extensive liver metastases (20, 21). This prompted our study to limit enrollment to those with a tumor burden in the liver of 25% or less. Additionally, our previous pre-clinical studies have shown that inclusion of a tumor associated antigen within VSV significantly enhances the CD8+ T cell mediated anti-tumor effects of oncolytic therapy by increasing the priming of CD8+ T cells against the tumor antigen (21, 22). Those studies also identified TYRP1 as a major melanoma associated antigen whose expression from the virus could stimulate anti-tumor immune responses (22). Therefore, in the current study we reasoned that incorporation of TYRP1 may focus the immune response more in the tumor, and less in off-target sites, while also improving T cell immunity.

### 2.2 Eligibility

We included adult patients with unresectable uveal melanoma who had an Eastern Cooperative Oncology Group (ECOG) performance status of 0 or 1 and a life expectancy ≥12 weeks. Patients were required to have measurable disease, a liver metastasis accessible for injection, and no more than 25% of the liver involved by malignancy. Patients with disease involving the brain or spinal cord were excluded. Patients could enroll in the first line setting or after prior treatments. See **Supplementary Materials (Supplementary Data 1.1)** for the full inclusion and exclusion criteria.

### 2.3 Study design

The study was a phase 1 clinical trial offered at Mayo Clinic in Rochester, MN. The primary objective was to determine the safety profile of VSV-IFNβ-TYRP1 administered via IV and IT routes. Secondary objectives were to gather preliminary data on tumor response and progression-free survival (PFS). We investigated both viral and immunological correlates.

Treatment was administered on Day 1 of Cycle 1. VSV-IFNβ-TYRP1 was injected into an accessible liver metastasis under image guidance (typically ultrasound). IV VSV-IFNβ-TYRP1 was given with 100ml of normal saline with 1% human serum albumin (HSA) over 30 minutes. To maximize patient safety, treatment was administered in an inpatient clinical research unit with close monitoring for 24-48 hours. Patients received acetaminophen 650 mg one hour prior to infusion, then every 6 hours for 24 hours to mitigate fever.

A 3 + 3 Phase I clinical trial design was carried out. The IT dose delivered was 3x10^7^ TCID_50_ (50% tissue culture infectious dose). IV doses started at dose level (DL) 1 1x10^10^ TCID_50_ then escalated to DL2 3x10^10^, DL3 1x10^11^, and DL4 3x10^11^. Both IT and IV doses were administered once. The clinical protocol was approved by the Mayo Clinic Institutional Review Board (IRB) and was conducted with Mayo Clinic IRB oversight.

### 2.4 Adverse event monitoring

Adverse event reporting was based on the Common Terminology Criteria for Adverse Events version 5 (CTCAE v5.0). Dose limiting toxicities (DLTs) were defined as: absolute neutrophil count (ANC) <500/mm^3^ for at least 7 days or in association with bacterial infection, platelet count <50,000/mm^3^ for a least 7 days or <25,000/mm^3^, creatinine >3x upper limit of normal, allergic reaction, autoimmune disorder, grade 3 or higher cytokine release syndrome (CRS)(graded based on 2014 Lee criteria), grade 3 viremia >24 hours, or other defined grade 3 events.

### 2.5 Response assessment

Response and progression were evaluated using a modified version of the new international criteria proposed by the revised Response Evaluation Criteria in Solid Tumors (RECIST) guidelines (version 1.1) (mRECIST). Disease progression for this protocol was defined as meeting the RECIST for disease progression on two consecutive evaluations at least 6 weeks apart. Patients were evaluated every 6 weeks until disease progression was confirmed on two subsequent scans.

### 2.6 Statistical analysis

The maximum tolerated dose was defined as the highest DL among those tested where at most 1 out of 6 patients developed a DLT, and the next highest DL was such that 2 of the 3 to 6 patients treated at this DL developed a DLT. Adverse events were summarized in frequency tables. Tumor responses were summarized by simple descriptive summary statistics. OS was defined as the time from registration to death due to any cause and was estimated with Kaplan-Meier (KM) curves.

### 2.7 Viral correlatives: pharmacokinetics and pharmacodynamics analyses

Pharmacokinetics and pharmacodynamics of VSV-IFNβ-TYRP1 were monitored by measuring VSV-nucleocapsid (N) RNA in whole blood by RT-qPCR and by analyzing plasma IFNβ levels using a IFNβ specific ELISA kit (PBL Assay Science, cat #41415, RRID : SCR_006852), respectively. Viral shedding into the buccal cavity and urine was analyzed using RT-qPCR for VSV-N RNA (23). Samples were also co-cultured on Vero cells for recovery of any infectious virus in buccal swabs and urine. Neutralizing anti-VSV antibody titers in the serum was determined using standard virus plaque reduction assay on BHK cells.

### 2.8 Immunologic correlatives: human T cell *in vitro* recall response assay

Peripheral blood mononuclear cells (PBMCs) were isolated from patient apheresis cones. Informed consent was obtained from all donors for the use of their sample for research purposes. CD3+ T cells were isolated using a magnetic sorting kit (Miltenyi Biotech) and activated using CD3/CD28 beads (ThermoFisher, cat #11161D, RRID : AB_2916088).

Autologous monocyte-derived dendritic cells were matured by isolating CD14+ cells via magnetic sorting (Miltenyi Biotech), followed by incubation with human GM-CSF (800 U/mL) and IL-4 (1000 U/mL). On Days 3 and 5, media was replaced with human GM-CSF (1600 U/mL) and IL-4 (1000 U/mL). On Day 7, non-adherent cells were collected, washed with PBS, and resuspended in medium containing GM-CSF (800 U/mL), IL-4 (1000 U/mL), TNF-alpha (1100 U/mL), IL-1beta (1870 U/mL), IL-6 (1000 U/mL), and PGE2 (1ug/mL).

Two days later, dendritic cells were either transfected with pCDNA3.1 expression plasmids (NO insert, TYRP1, GP100 or OVA) (1µg/10^6^ DC) or were pulsed with peptide libraries of VSV, hTERT, Cyclin D1 or with OVA-derived SIINFEKL peptide (5µg/ml). 10^6^ Pre-activated, T cells were then co-cultured at a ratio of 10:1 with the transfected or pulsed CD14+ *in vitro* matured dendritic cells (10^5^ cells per well where possible) prepared from the same donor in interferon gamma ELIspot wells. Three days later, wells were developed, and spots were counted.

T cell responses to antigens of interest were defined as 1) at least a doubling of the number of spots formed in post-treatment versus pre-treatment, and 2) no overlap of the 95% confidence intervals of pre- and post-treatment responses.

## 3 Results

### 3.1 Patient characteristics

12 patients with previously treated MUM were enrolled from July 2019 to January 2022. Three patients were treated at each DL (1–4). Since we did not see objective evidence of clinical efficacy amongst the first 12 patients on study, the potential benefit of enrolling an additional 3 patients was not deemed worth the risk. The median age was 69.5 years. 5 patients were female (41.7%) and 7 were male (58.3%). All were diagnosed with MUM involving the liver. 1 patient had a prior malignancy, which was a hormone-producing pituitary tumor that had since been resected. Patients received a median of one line of prior systemic therapy (range 0-2). Prior treatments included: ipilimumab/nivolumab, pembrolizumab, nivolumab, carboplatin/paclitaxel/pembrolizumab, and trametinib. ECOG performance status was either 0 (91.7%) or 1 (8.3%). Additional details of baseline patient characteristics can be found in **Table 1**.

**Table 1:** Patient characteristics.

	Total (N=12)
Age	
Mean (SD)	66.8 (8.59)
Median	69.5
Range	49.0, 78.0
Gender, n (%)	
Female	5 (41.7%)
Male	7 (58.3%)
Lines of prior systemic therapy, n (%)	
2	4 (33.3%)
1	6 (50.0%)
0	2 (16.7%)
Prior exposure to ICI, n (%)*	
Yes	10 (83.3%)
No	2 (16.7%)
ECOG Performance Status, n (%)	
0	11 (91.7%)
1	1 (8.3%)

* Includes ipilimumab/nivolumab, pembrolizumab, nivolumab. Overview of demographics and prior therapies for the 12 patients enrolled in the trial.

### 3.2 Adverse events

**Figure 1:**
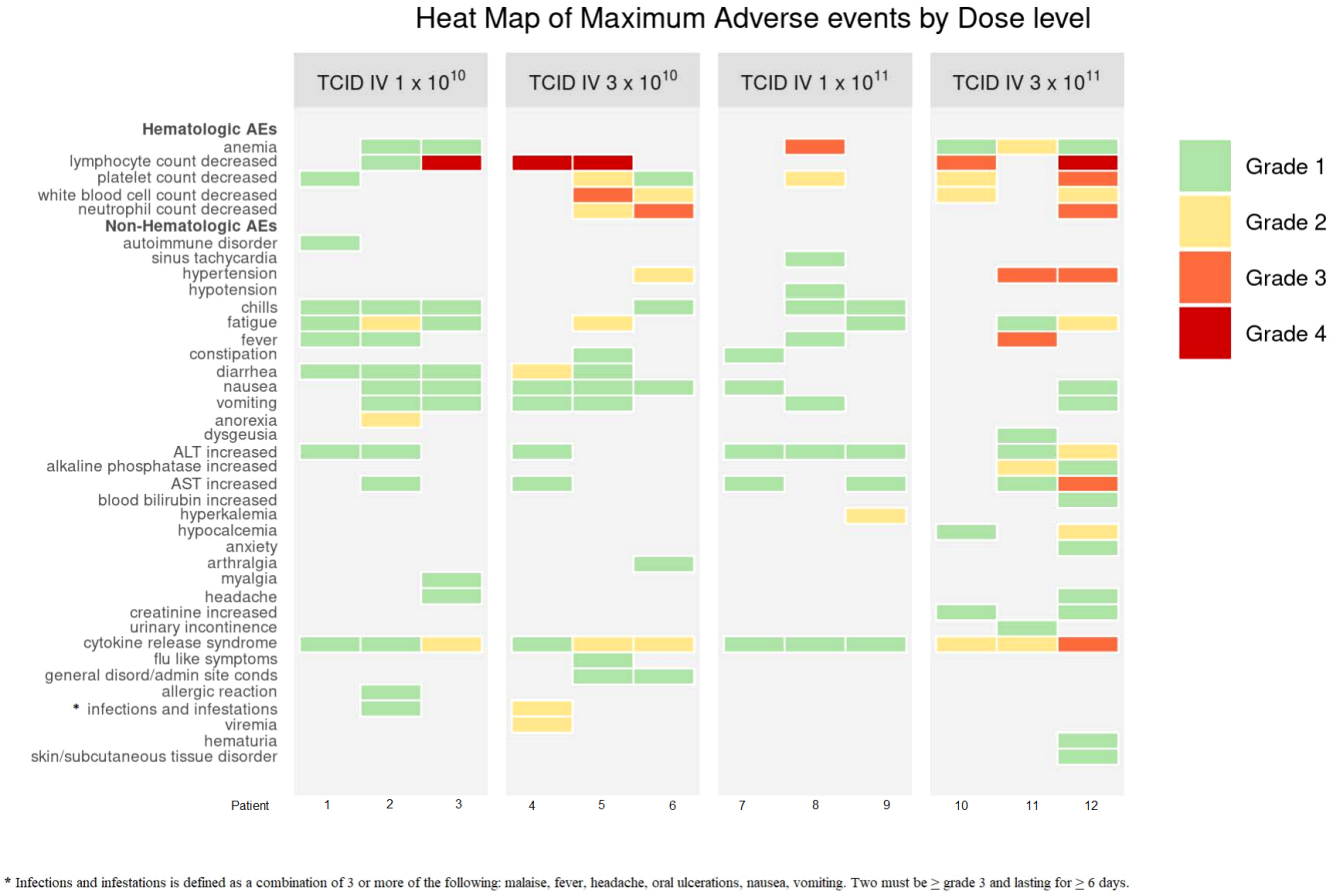
Heat Map of Maximum Adverse Events by Dose Level. Dose limiting toxicities (DLTs) occurred at DL4 for 1 patient (A4.3). These included: decreased platelets (grade 3), increased AST (grade 3), CRS (grade 3). CRS criteria met due to organ toxicity of grade 3 hepatitis. The DLTs resolved within 14 days. Non-DLT AEs did not significantly increase with dose escalation. * Infections and infestations is defined as a condition.

For DLs 1-2, common AEs were fatigue, hematological toxicities (decreased platelets, total white blood cells, lymphocytes, neutrophils), and CRS (grade 1-2). 1 patient experienced a VSV infection at DL2, seen as fevers/chills, fatigue, nausea/vomiting, diarrhea for ≥6 days. For DL3, common AEs were CRS (grade 1), hematological (decreased platelets and hemoglobin), increased liver function tests (LFTs), including increased AST and alanine aminotransferase (ALT). At DL4, AEs included fatigue, fever, CRS (grade 2-3), hematological toxicities (decreased platelets and lymphocytes), and AST elevation (**Figure 1**). AEs did not significantly increase with dose escalation.

1 patient at DL4 experienced DLTs. These included: decreased platelet count (grade 3), increased aspartate aminotransferase (AST) (grade 3), and CRS (grade 3). CRS criteria was met due to organ toxicity of CTCAE grade 3 hepatitis. 3 patients in total were entered at DL4 with at least 14 days of observation for each patient prior to enrollment of the next patient.

### 3.3 Efficacy

**Figure 2:**
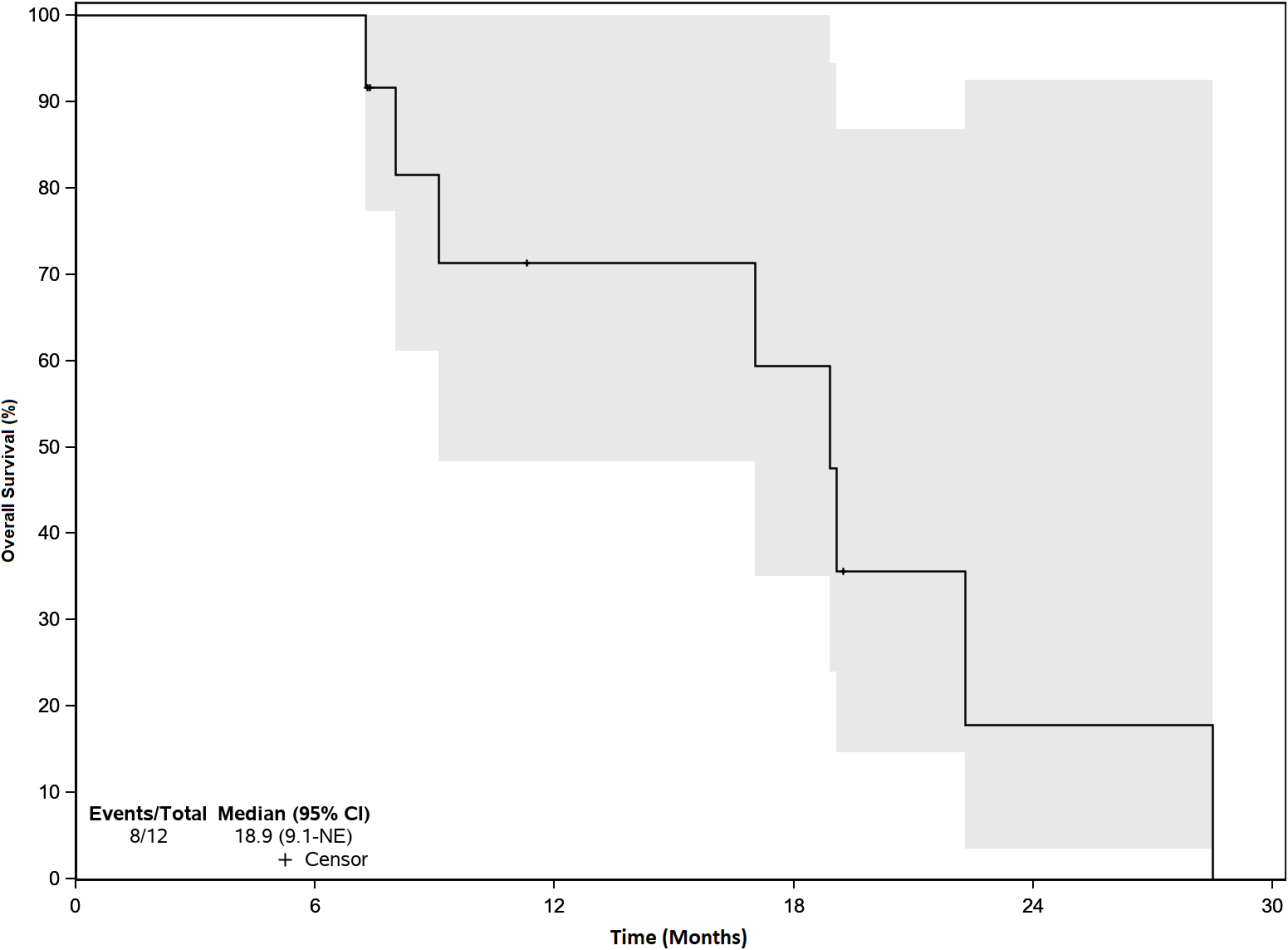
Overall Survival. Median Overall Survival (OS) was 18.9 months. There were no objective responses, but efficacy was a secondary endpoint. 4 patients had stable disease and 8 patients had progressive disease. The gray zone around the curve represents the 95% confidence interval (CI).

There were no objective responses to VSV-IFNβ-TYRP1 therapy. 4 patients had stable disease (SD) and 8 had progressive disease (PD) as their best response. Of the patients with initial stable disease, 1 progressed at the subsequent scan (3 months after starting treatment), and 3 patients were censored shortly after imaging because they started another line of therapy due to treating clinician discretion despite lack of progression. Next treatments after VSV-IFNβ-TYRP1 included: ipilimumab/nivolumab (6), nivolumab/cabozantinib (1), tebentafusp (1), chemotherapy (3), and liver-directed radiation (1). Of the 8 patients who received ICI therapy immediately after VSV-IFNβ-TYRP1 therapy, 1 had an unconfirmed partial response (PR) after 4 cycles, then later had PD after 9 months, 1 had initial SD and then had PD after 5 months, and 6 had PD as their best response. Median follow up was 19.1 months. Median OS was 18.9 months (**Figure 2**).

### 3.4 Kinetics of VSV-IFβ-TYRP1 infection and anti-VSV antibody response

**Figure 3:**
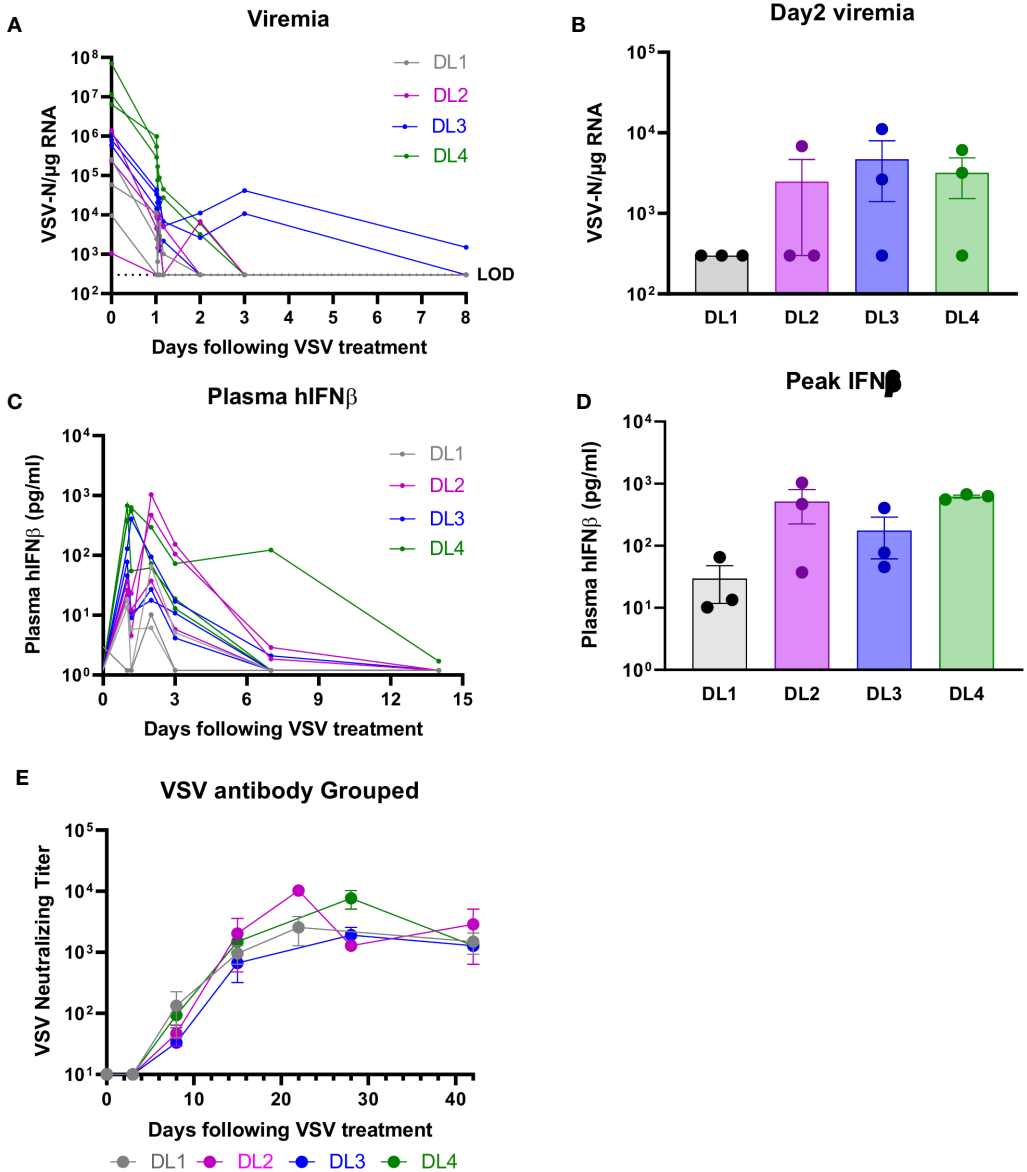
Viral Correlatives. **(A, B)** VSV-nucleocapsid (N) RNA was below level of detection (LOD) by day 3 (except for DL3, which was detected on day 8). VSV-N was detected only in mouth wash, no other body fluids (see **supplementary data**). **(C, D)** IFN-β peak levels were lowest for DL1 and similar at DL2-4. **(E)** Neutralizing antibody titers were across all DLs.

Viremia was measured as VSV-N RNA in blood at the end of the infusion, 30 and 60 minutes, 2 and 4 hours, and days 2, 3, 8, and 15. **Figure 3A** demonstrates that at all DLs, except for DL3, VSV-N RNA was highest at the end of the infusion and was below the level of detection (LOD) by day 3. For DL3, VSV-N RNA continued to be detected on day 8. Day 2 viremia was similar across DL2-4 and was lowest for DL1 (**Figure 3B**). For viral shedding, VSV-N was detected only in the mouth wash at DL1 (Days 2 and 8) and DL3 (Day 2) (**Supplementary Figure 1**). VSV-N was not found in urine or on buccal swabs. VSV neutralizing antibody titers generally peaked by week 2 and were similar between all DLs by day 28 (**Figure 3E**). Plasma IFN-β peak levels were lowest for DL1 and similar at DL2-4 (**Figure 3D**). Plasma IFN-β levels were higher for DL4 at Day 7 compared to the other DLs, but later decreased to a similar level as the other DLs by Day 14 (**Figure 3C**).

### 3.5 Emergence of T cell responses to TYRP1 and other melanoma antigens

**Figure 4:**
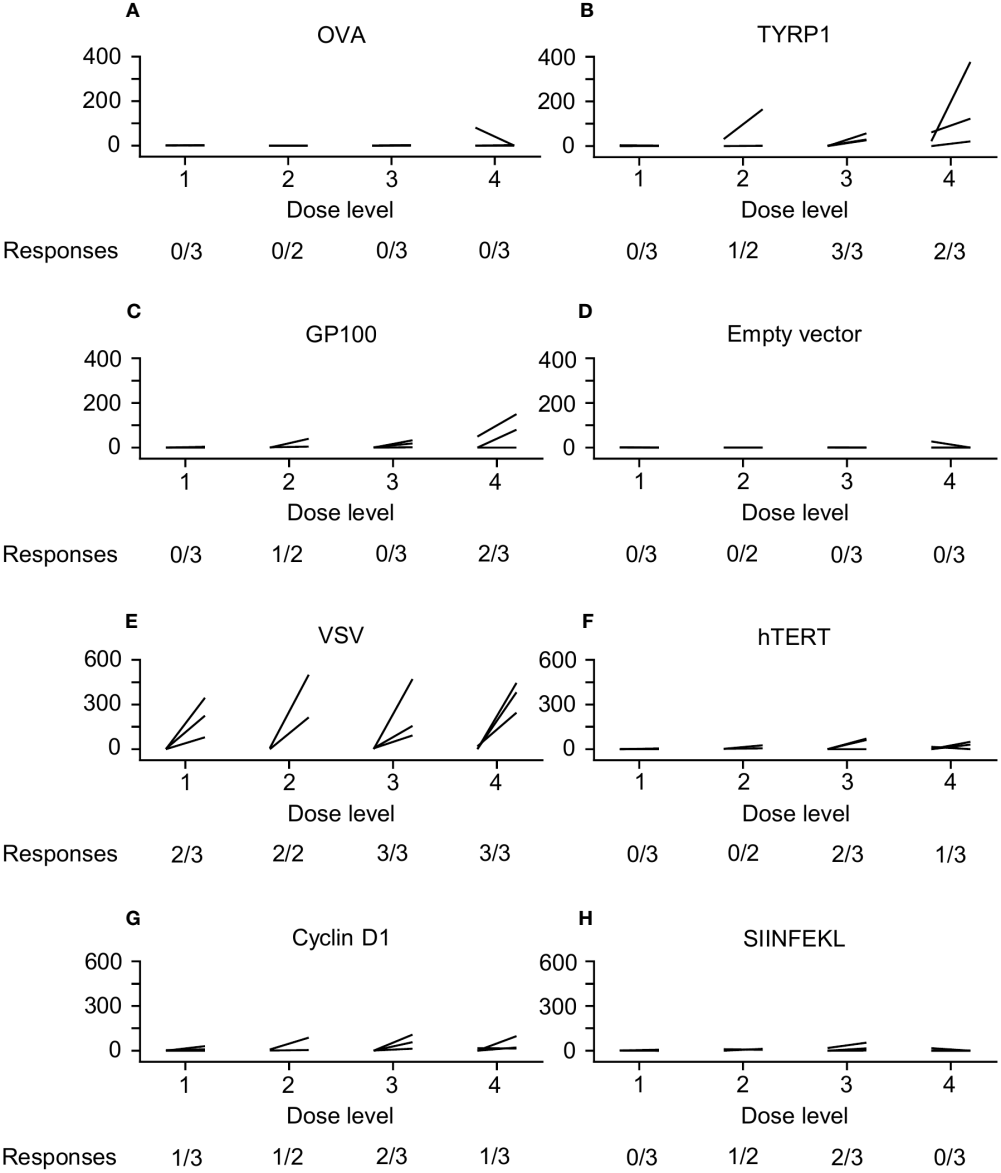
ELIspot Data. CD8+ T cells and dendritic cells (DCs) were isolated from peripheral blood mononuclear cells (PBMCs). The DCs were then transfected with plasmids driving expression of OVA (positive control), TYRP1 (vaccine antigen), gp100 (another melanocyte differentiation antigen), or nothing (negative control); then cultured with purified CD8+ T cells **(A–D)**. Alternatively, PBMCs were cultured with peptide pools from VSV, hTERT (melanoma antigen), Cyclin D1 (melanoma antigen), or the peptide SIINFEKL (irrelevant antigen) **(E–H)**. T cell responses to VSV were seen in 10/11 patients across all DLs. Responses to TYRP-1 were seen more frequently in patients treated on DL3 and DL4 (5/6 patients versus 1/5 patients on DL1 and DL2). T cell responses were induced to other melanoma antigens, including hTERT (3 patients), gp100 (3 patients), and Cyclin D1 (5 patients).

CD8+ T cells and CD14+ monocytes were isolated from PBMCs. The monocytes were cultured with IL-4 and GM-CSF to produce DCs, which were then transfected with plasmids (1µg/10^6^ DC) driving expression of OVA (positive control), TYRP1 (vaccine antigen), gp100 (another melanocyte differentiation antigen), or nothing (negative control). 10^5^ DCs were then cultured with 10^6^ purified CD8+ T cells (**Figures 4A-D**). Alternatively, 10^5^ untransfected DCs were cultured with peptide pools (5µg/ml) from VSV, hTERT (melanoma antigen), Cyclin D1 (melanoma antigen), or the peptide SIINFEKL (irrelevant antigen) for 24 hours and then 10^6^ purified CD8+ T cells were added in interferon gamma ELISPOT wells. Three days later, wells were developed and spots counted (**Figures 4E-H**). Of 12 patients, 11 had sufficient PBMCs at baseline and at least 1 post-treatment time point (typically Day 42). While T cell responses to VSV were seen in 10/11 patients across all DLs, responses to TYRP-1 were seen more frequently in patients treated on DLs 3 and 4 (5 of 6 patients) versus DLs 1 and 2 (1 of 5 patients). T cell responses were induced to other melanoma antigens, including hTERT, gp100, and Cyclin D1 in some patients (**Figure 4**).

## 4 Discussion

Our study evaluated VSV-IFNβ-TYRP1 given via IT and IV administration in a previously treated population of patients with MUM. Treatment was generally well tolerated with 1 patient experiencing DLTs at DL4, which resolved in 14 days. DLTs included: decreased platelets (grade 3), increased AST (grade 3), and CRS (grade 3). CRS criteria was met due to organ toxicity seen as grade 3 hepatitis. For our primary endpoint of safety, VSV-IFNβ-TYRP1 highlights that a VSV-IFNβ OV can be safely administered concurrently with IT injection and IV administration if the correct precautions are taken. A prior study of VSV-IFNβ (NCT01628640) resulted in a patient death after IT injection, which is thought to be due to extensive liver metastases. This prompted our study to limit enrollment to patients with a tumor burden in the liver of 25% or less. Additionally, incorporation of TYRP1 may focus the immune response more in the tumor, and less in off-target sites, while also improving T cell immunity. Combining IT and IV administration has not been previously investigated. In theory, this approach allows for direct introduction of the OV to the tumor to initiate a UM-specific immune response while the IV dose allows for a systemic exposure of the OV to other metastases. The robust anti-viral responses and emergence of T cell responses to melanoma antigens seen here lends support to this hypothesis. However, we recognize that biodistribution of IV therapies tends to be limited by dilution in systemic circulation and sequestration by the reticuloendothelial system (15). We opted for single doses of both IT and IV VSV-IFNβ-TYRP1 since repeat dosing could lead to induction of VSV immunity, and thus viral clearance. Additionally, repeated IT dosing to liver lesions would pose risk to the patient.

While there were no clear objective responses to VSV-IFNβ-TYRP1, efficacy was a secondary endpoint. However, we did find dose-dependent immunogenicity to virus encoded TYRP1, with more responses at DL 3 and 4 compared to DL 1 and 2 (**Figure 4**). Additionally, after treatment with VSV-IFNβ-TYRP1, 2 of 8 patients who were subsequently treated with ICIs found clinical benefit of prolonged SD for >6 months, which is longer than the median PFS reported in the trials for both single and dual immune checkpoint inhibition (1, 24–27). Our ELIspot data provides insight into the sustained response to ICIs for these 2 patients. Two patients at DL4 had significant responses to gp100, a well-documented melanoma associated tumor antigen (**Figure 4**). A patient at DL2 showed a lesser, but significant, T cell response to gp100. This may indicate epitope spreading against melanoma-relevant antigens, which could increase response to other forms of immunotherapy (28). Most interestingly, the 3 patients responding to gp100 received ICI as the next line of therapy, which led to SD in the aforementioned 2 patients, lasting for 22 and 36 weeks (**Table 2**).

**Table 2:** Induced Immune Response to Melanoma Antigens.

Patient	Dose Level	Response to TYRP1	Response to gp100	Response to hTERT	Response to Cyclin D1
6	2	Yes	Yes	No	Yes
7	3	Yes	No	Yes	Yes
All others	all	3/9	2/9	2/9	3/9

2 patients (6 and 7) treated with ICIs after the trial experienced clinical benefit, seen as stable disease (SD) for 22 and 36 weeks, respectively. These patients showed clear T cell reactivity against both virus encoded TYRP1 and other melanoma associated antigens (gp100, hTERT, cyclin D1), which suggests increased immunogenicity after VSV-IFNβ-TYRP1.

As well as an empty vector for re-stimulation of T cells, we used the SIINFEKL peptide from ovalbumin (which is a known H2Kb-restricted murine epitope) as a negative control for stimulation of T cells. Since this is a known epitope in the C57Bl/6 murine system, we reasoned that this would form a negative control to identify any background T cell reactivity against an irrelevant peptide which could be induced by the presence of generalized T cell stimulation by VSV, an effect we have observed in some pre-clinical studies. In this respect, we did observe small but significant increases in T cell reactivity to this non-specific SIINFEK stimulator in 3 patients at DLs 2 and 3, indicating hyper-stimulation of T cells by VSV infection. Interestingly, at DLs 3 and 4 we also observed T cell reactivity against hTERT and, at all dose levels, against Cyclin D1. Given the evidence of a possible VSV-associated enhanced T cell reactivity against SIINFEKL, it is difficult to interpret the significance of the responses to hTERT or Cyclin D1 as they might reflect epitope spreading against possible tumor associated antigens.

Finally, as expected, we observed robust anti-VSV T cell responses in the majority of patients treated with the virus (**Figure 4**). We hypothesize that the strength, and reproducibility, of these anti-viral T cell responses are attributable to IV administration of virus leading to direct lymph node access. We have observed in pre-clinical models that the anti-VSV response is considerably enhanced by IV as opposed to IT delivery (unpublished). Additionally, there is a trend towards increased peak levels of IFNß detectable in the plasma with the transition from DL1 to DLs 2-4 and an apparent prolonged level of circulating IFNß at the highest dose level 4 (**Figures 3C, D**). Our rationale for inclusion of the IFNß gene in the virus was both to enhance safety by shutting down of viral replication in off target non tumor tissues, and to provide further immune stimulatory signals for the activation of anti-tumor T cell responses. It is tempting to speculate that the combination of 1) the higher doses of the virus (DLs 3&4), 2) the subsequent higher levels of TYRP1 antigen released and 3) the prolonged levels of immune stimulation provided by IFNß persistence at these dose levels combine to generate the epitope spreading against gp100 seen in the two patients who subsequently responded well to ICIs. Further trials will be needed to test this hypothesis. Therefore, overall we conclude that, although response to our OV alone is not sufficient for clinical benefit, the clinical outcomes for at least two patients to ICIs, coupled to their T cell responses showing potential epitope spreading, are encouraging.

In our pre-clinical melanoma models, we observed that anti-PD1 therapy administered concomitantly with VSV virotherapy significantly decreased anti-tumor T cell responses (as measured by IL-12 levels). In contrast, when anti-PD1 therapy is administered sequentially to viral injection, anti-tumor immune T cell responses increase (29). These and other studies from our laboratory (data not shown, Kendall et al. in preparation), indicate that ICIs can work on both anti-viral and anti-tumor T cells; therefore, optimal enhancement of the anti-tumor T cell response by ICIs may require a clear temporal separation of the virus and ICI administration to allow the reinvigoration of anti-tumor T cell response preferentially over the anti-viral response. The 2 patients in our study that benefitted from ICI therapy after VSV-IFNβ-TYRP1 further supports these findings both clinically and in their T cell reactivity (**Table 2**). Our observations suggest that virotherapy led to epitope spreading in which T cell responses against additional tumor associated antigens was induced, and that VSV-IFNβ-TYRP1 may induce some level of anti-tumor T cell response which are then susceptible to later reinvigoration with ICIs (30). Further studies with larger numbers of patients will be required to confirm such a hypothesis.

While T-VEC is the only FDA approved OV to date, as of 2020 there have been 30 trials performed investigating OVs in melanoma (14, 15). The limited efficacy of OVs thus far may be related to an imbalance between viral replication and host immune response, inadequate tumor specificity, neutralizing antibodies, an immunosuppressive tumor-immune microenvironment (TIME), and differences in the microbiome (15, 30). We theorized that our OV would overcome some of these barriers by incorporation of TYRP1 to improve UM specificity and immunogenicity, and IFNβ to enhance T cell responses. Other factors, such as the immunosuppressive TIME were not accounted for in our study, and may be modulated by incorporating additional therapies, such as ICIs. After OV-induced oncolysis and immune infiltration, ICIs may have additional neoantigens (or non-mutated antigens such as TYRP1) available for a more robust tumor-specific immune response (31).

Looking forward, we propose combining OVs, such as VSV-IFNβ-TYRP1, with both additional target antigens to avoid the chances of antigen escape, as well as with other immunomodulatory treatments given the evidence of epitope spreading observed (32). There are multiple pre-clinical studies and a few clinical studies to support the combination of OVs and immunotherapy to improve outcomes. A phase 1b trial of T-VEC in combination with pembrolizumabresulted in an objective response rate of 62% (33). A preclinical study on VSV-IFN-NIS in combination with an anti-PDL-1 antibodyresulted in superior survival in mice with C1498AML tumors (34). Hence, we are keen to eventually use VSV-IFNβ-TYRP1 in combination with other therapies to enhance clinical outcomes.

Overall, VSV-IFNβ-TYRP1 is safe when given via IT injection and IV administration in patients who have less than 25% tumor burden in the liver. While there were no clear objective responses to VSV-IFNβ-TYRP1, dose-dependent immunogenicity to melanoma antigens was seen, both to virus-encoded TYRP1 and other melanoma antigens (gp100, hTERT, and cyclin D1), suggesting epitope spreading. Subsequent ICI therapy led to sustained responses for 2 patients, whom both showed evidence of epitope spreading on T cell *in vitro* recall reponse assays. Future studies will focus on VSV-IFNβ-TYRP1 in combination with other therapies to improve efficacy.

## Data availability statement

The original contributions presented in the study are included in the article/**Supplementary Material**. Further inquiries can be directed to the corresponding author.

## Ethics statement

The studies involving humans were approved by Mayo Clinic Institutional Review Board. The studies were conducted in accordance with the local legislation and institutional requirements. The participants provided their written informed consent to participate in this study. Written informed consent was obtained from the individual(s) for the publication of any potentially identifiable images or data included in this article.

## Author contributions

KS: Writing – original draft, Writing – review & editing, Project administration. KP: Conceptualization, Formal Analysis, Investigation, Methodology, Visualization, Writing – review & editing. JP: Conceptualization, Writing – review & editing. AW: Investigation, Writing – review & editing. CS: Data curation, Formal Analysis, Visualization, Writing – review & editing. JA: Data curation, Formal Analysis, Writing – review & editing. AN: Investigation, Writing – review & editing. LZ: Investigation, Writing – review & editing. NP: Investigation, Writing – review & editing. TK: Investigation, Writing – review & editing. JT: Investigation, Writing – review & editing. MM: Investigation, Writing – review & editing. HM: Resources, Writing – review & editing. LK: Resources, Writing – review & editing. RM: Resources, Writing – review & editing. AZD: Resources, Writing – review & editing. YY: Resources, Writing – review & editing. AD: Resources, Writing – review & editing. SM: Resources, Writing – review & editing. MF: Investigation, Writing – review & editing. RV: Conceptualization, Formal Analysis, Investigation, Methodology, Visualization, Writing – review & editing. RD: Conceptualization, Supervision, Writing – review & editing. MB: Conceptualization, Project administration, Writing - original draft, Writing - review & editing.

## References

[1] SEER*Explorer. Melanoma of the skin, Recent Trends in SEER Relative Survival Rate . Available at: https://seer.cancer.gov/statistics-network/explorer/application.html?site=53&data_type=4&graph_type=2&compareBy=stage&chk_stage_106=106&relative_survival_interval=5&sex=1&race=1&age_range=1&advopt_precision=1&advopt_show_ci=on&hdn_view=0&advopt_display=2#graphArea (Accessed September 16 2022).

[2] Swetter SM Thompson JA Albertini MR Barker CA Baumgartner J Boland G. NCCN guidelines insights: melanoma: cutaneous, version 2.2021. J Natl Compr Canc Netw (2021) 19(4):364–76. doi: 10.6004/jnccn.2021.0018 33845460

[3] Switzer B Puzanov I Skitzki JJ Hamad L Ernstoff MS. Managing metastatic melanoma in 2022: A clinical review. JCO Oncol Pract (2022) 18(5):335–51. doi: 10.1200/OP.21.00686 PMC9810138 35133862

[4] Tawbi HA SChadendorf D Lipson EJ Ascierto PA Matamala L Castillo Gutiérrez E. Relatlimab and nivolumab versus nivolumab in untreated advanced melanoma. N Engl J Med (2022) 386(1):24–34. doi: 10.1056/NEJMoa2109970 34986285 PMC9844513

[5] Xu Y Lou L Wang Y Miao Q Jin K Chen M. Epidemiological study of uveal melanoma from US surveillance, epidemiology, and end results program (2010-2015). J Ophthalmol (2020) 2020:3614039. doi: 10.1155/2020/3614039 32148939 PMC7049826

[6] Krantz BA Dave N Komatsubara KM Marr BP Carvajal RD. Uveal melanoma: epidemiology, etiology, and treatment of primary disease. Clin Ophthalmol (2017) 11:279–89. doi: 10.2147/OPTH.S89591 PMC5298817 28203054

[7] Piulats JM Shoushtari AN Ochsenreither S Abdullah SE Holland C McCully ML. Overall survival (OS) in metastatic uveal melanoma: a summary of recent prospective trials. ASCO Annual Meeting; 2022 June 2; Chicago, IL, USA. J Clin Oncol (2022) 40(16):e21598–8. doi: 10.1200/JCO.2022.40.16_suppl.e21598

[8] Wolchok JD Chiarion-Sileni V Gonzalez R Grob JJ Rutkowski P Lao CD. Long-term outcomes with nivolumab plus ipilimumab or nivolumab alone versus ipilimumab in patients with advanced melanoma. J Clin Oncol (2022) 40(2):127–37. doi: 10.1200/JCO.21.02229 PMC8718224 34818112

[9] Long GV Schachter J Arance A Grob JJ Mortier L Daud A. Long-term survival from pembrolizumab (pembro) completion and pembro retreatment: Phase III KEYNOTE-006 in advanced melanoma. ASCO Annual Meeting; 2020 May 25; Chicago, IL, USA. J Clin Oncol (2020) 38:15. doi: 10.1200/JCO.2020.38.15_suppl.10013

[10] Hoefsmit EP Rozeman EA Van TM Dimitriadis P Krijgsman O Conway JW. Comprehensive analysis of cutaneous and uveal melanoma liver metastases. J Immunother Cancer (2020) 8(2):e001501. doi: 10.1136/jitc-2020-001501 33262254 PMC7713183

[11] Hilke FJ Sinnberg T Gschwind A Niessner H Demidov G Amaral T. Distinct mutation patterns reveal melanoma subtypes and influence immunotherapy response in advanced melanoma patients. Cancers (Basel) (2020) 12(9):2359. doi: 10.3390/cancers12092359 32825510 PMC7563780

[12] Bakhoum MF Esmaeli B. Molecular characteristics of uveal melanoma: insights from the cancer genome atlas (TCGA) project. Cancers (Basel) (2019) 11(8):1061. doi: 10.3390/cancers11081061 31357599 PMC6721321

[13] Nathan P Hassel JC Rutkowski P Baurain JF Butler MO Schlaak M. Overall survival benefit with tebentafusp in metastatic uveal melanoma. N Engl J Med (2021) 385(13):1196–206. doi: 10.1056/NEJMoa2103485 34551229

[14] Andtbacka RH Kaufman HL Collichio F Amatruda T Senzer N Chesney J. Talimogene laherparepvec improves durable response rate in patients with advanced melanoma. J Clin Oncol (2015) 33(25):2780–8. doi: 10.1200/JCO.2014.58.3377 26014293

[15] Macedo N Miller DM Haq R Kaufman HL. Clinical landscape of oncolytic virus research in 2020. J Immunother Cancer (2020) 8(2):e001486. doi: 10.1136/jitc-2020-001486 33046622 PMC7552841

[16] Cook J Peng KW Witzig TE Broski SM Villasboas JC Paludo J. Clinical activity of single-dose systemic oncolytic VSV virotherapy in patients with relapsed refractory T-cell lymphoma. Blood Adv (2022) 6(11):3268–79. doi: 10.1182/bloodadvances.2021006631 PMC9198941 35175355

[17] Obuchi M Fernandez M Barber GN. Development of recombinant vesicular stomatitis viruses that exploit defects in host defense to augment specific oncolytic activity. J Virol (2003) 77(16):8843–56. doi: 10.1128/jvi.77.16.8843-8856.2003 PMC167243 12885903

[18] Medrano RFV Hunger A Mendonça SA Barbuto JAM Strauss BE. Immunomodulatory and antitumor effects of type I interferons and their application in cancer therapy. Oncotarget (2017) 8(41):71249–84. doi: 10.18632/oncotarget.19531 PMC5642635 29050360

[19] de Vries TJ Trancikova D Ruiter DJ van Muijen GN. High expression of immunotherapy candidate proteins gp100, MART-1, tyrosinase and TRP-1 in uveal melanoma. Br J Cancer (1998) 78(9):1156–61. doi: 10.1038/bjc.1998.646 PMC2063001 9820172

[20] Jenks N Myers R Greiner SM Thompson J Mader EK Greenslade A. Safety studies on intrahepatic or intratumoral injection of oncolytic vesicular stomatitis virus expressing interferon-beta in rodents and nonhuman primates. Hum Gene Ther (2010) 21(4):451–62. doi: 10.1089/hum.2009.111 PMC2865219 19911974

[21] Willmon CL Saloura V Fridlender ZG Wongthida P Diaz RM Thompson J. Expression of IFN-beta enhances both efficacy and safety of oncolytic vesicular stomatitis virus for therapy of mesothelioma. Cancer Res (2009) 69(19):7713–20. doi: 10.1158/0008-5472.CAN-09-1013 PMC3891512 19773437

[22] Diaz RM Galivo F Kottke T Wongthida P Qiao J Thompson J. Oncolytic immunovirotherapy for melanoma using vesicular stomatitis virus. Cancer Res (2007) 67(6):2840–8. doi: 10.1158/0008-5472.CAN-06-3974 17363607

[23] Zhang L Steele MB Jenks N Grell J Suksanpaisan L Naik S. Safety studies in tumor and non-tumor-bearing mice in support of clinical trials using oncolytic VSV-IFNβ-NIS. Hum Gene Ther Clin Dev (2016) 27(3):111–22. doi: 10.1089/humc.2016.061 PMC5076487 27532609

[24] Kottschade LA McWilliams RR Markovic SN Block MS Villasboas Bisneto J Pham AQ. The use of pembrolizumab for the treatment of metastatic uveal melanoma. Melanoma Res (2016) 26(3):300–3. doi: 10.1097/CMR.0000000000000242 26848796

[25] Algazi AP Tsai KK Shoushtari AN Munhoz RR Eroglu Z Piulats JM. Clinical outcomes in metastatic uveal melanoma treated with PD-1 and PD-L1 antibodies. Cancer (2016) 122(21):3344–53. doi: 10.1002/cncr.30258 PMC5767160 27533448

[26] Pelster MS Gruschkus SK Bassett R Gombos DS Shephard M Posada L. Nivolumab and ipilimumab in metastatic uveal melanoma: results from a single-arm phase II study. J Clin Oncol (2021) 39(6):599–607. doi: 10.1200/JCO.20.00605 33125309 PMC8257877

[27] Piulats JM Espinosa E de la Cruz Merino L Varela M Alonso Carrión L Martín-Algarra S. Nivolumab plus ipilimumab for treatment-naïve metastatic uveal melanoma: an open-label, multicenter, phase II trial by the spanish multidisciplinary melanoma group (GEM-1402). J Clin Oncol (2021) 39(6):586–98. doi: 10.1200/JCO.20.00550 33417511

[28] Brossart P. The role of antigen spreading in the efficacy of immunotherapies. Clin Cancer Res (2020) 26(17):4442–7. doi: 10.1158/1078-0432.CCR-20-0305 32357962

[29] Kottke T Tonne J Evgin L Driscoll CB van Vloten J Jennings VA. Oncolytic virotherapy induced CSDE1 neo-antigenesis restricts VSV replication but can be targeted by immunotherapy. Nat Commun (2021) 12(1):1930. doi: 10.1038/s41467-021-22115-1 33772027 PMC7997928

[30] Moaven O Mangieri CW Stauffer JA Anastasiadis PZ Borad MJ. Strategies to develop potent oncolytic viruses and enhance their therapeutic efficacy. JCO Precis Oncol (2021) 5. doi: 10.1200/PO.21.00003 PMC8232397 34250395

[31] Cerqueira OLD Antunes F Assis NG Cardoso EC Clavijo-Salomón MA Domingues AC. Perspectives for combining viral oncolysis with additional immunotherapies for the treatment of melanoma. Front Mol Biosci (2022) 9:777775. doi: 10.3389/fmolb.2022.777775 35495634 PMC9048901

[32] Pulido J Kottke T Thompson J Galivo F Wongthida P Diaz RM. Using virally expressed melanoma cDNA libraries to identify tumor-associated antigens that cure melanoma. Nat Biotechnol (2012) 30(4):337–43. doi: 10.1038/nbt.2157 PMC3891505 22426030

[33] Ribas A Dummer R Puzanov I VanderWalde A Andtbacka RHI Michielin O. Oncolytic virotherapy promotes intratumoral T cell infiltration and improves anti-PD-1 immunotherapy. Cell (2017) 170(6):1109–1119.e10. doi: 10.1016/j.cell.2017.08.027 28886381 PMC8034392

[34] Shen W Patnaik MM Ruiz A Russell SJ Peng KW. Immunovirotherapy with vesicular stomatitis virus and PD-L1 blockade enhances therapeutic outcome in murine acute myeloid leukemia. Blood (2016) 127(11):1449–58. doi: 10.1182/blood-2015-06-652503 PMC4797021 26712908

