# Supplementary material for: A phase I oncolytic virus trial with vesicular stomatitis virus expressing human interferon beta and tyrosinase related protein 1 administered intratumorally and intravenously in uveal melanoma: safety, efficacy, and T cell responses
Source: Front Immunol. 2023 Oct 31;14:1279387. doi: 10.3389/fimmu.2023.1279387 (PMC10644866; doi:10.3389/fimmu.2023.1279387)
Supplement: Supplementary file 1 [file DataSheet_1.docx]

Supplementary Material

# Supplementary Data

## Eligibility Criteria

Inclusion Criteria

- Age ≥18 years
- Histologically or cytologically confirmed diagnosis of unresectable Stage III or metastatic (Stage IV) melanoma, including metastatic ocular melanoma.
- Treatment is permitted to be treated in the first line setting.
- Measurable disease by any imaging modality as defined by RECIST (version 1.1)
- Injectable disease (i.e., suitable for direct injection or through the use of ultrasound guidance) defined as at least one safely accessible liver metastasis for patients with metastatic ocular melanoma
- No more than 25% overall tumor involvement of the liver by MRI imaging
- Child Pugh Score A
- Absence of ascites
- No portal vein thrombosis
- Have resolution of all previous treatment-related toxicities to Grade 1 severity or lower
- Life expectancy of ≥12 weeks
- ECOG Performance Status (PS) 0 or 1
- Willing and have the ability to comply with scheduled visits (including geographical proximity), treatment plans, laboratory tests, and other study procedures
- Willing to provide all biological specimens as required by the protocol Including fresh tissue for biomarker analysis (metastatic melanoma cohort with accessible injectable lesions only).
- Negative pregnancy test done ≤7 days prior to registration, for persons of childbearing potential only NOTE: If the urine test is positive or cannot be confirmed as negative, a serum pregnancy test will be required
- Willing to use an adequate method of contraception from the first dose of study medication through 120 days after the last dose of study medication, for persons of childbearing potential or persons able to father a child only
- The following laboratory values obtained ≤14 days prior to registration:
- Absolute neutrophil count (ANC) ≥1500/mm3
- Platelet count ≥100,000/mm3
- Hemoglobin ≥9.0 g/dL (without need for hematopoietic growth factor or transfusion support)
- Alanine aminotransferase (ALT) ≤2.5 x ULN
- Aspartate transaminase (AST) ≤2.5 x ULN
- Total bilirubin ≤1.5 x ULN
- PT ≤1.5 x ULN (or international normalization ratio [INR] ≤1.4) or
- PTT/aPTT ≤ULN
- Serum creatinine within institutional limits of normal (≤ULN)

Exclusion Criteria

- Known standard therapy for the patient’s disease that is potentially curative or definitely capable of extending life expectancy
- Need for concurrent chemotherapy, immunotherapy, radiotherapy, ablation therapy or any ancillary therapy considered investigational (used for a non-FDA approved indication or in the context of a research investigation)
- Any of the following prior therapies:
  - Prior chemotherapy ≤2 weeks prior to registration
  - Prior immunotherapy (monoclonal antibodies) ≤3 weeks prior to registration
  - Prior experimental agent ≤2 weeks prior to registration
  - Prior radiation therapy ≤2 weeks prior to registration
- Prior non-oncology vaccine therapies used for the prevention of infectious disease ≤28 days prior to registration
- Requires concomitant treatment with therapeutic anticoagulants
- Minor surgical or interventional procedure ≤7 days prior to registration
- Major surgical procedure ≤21 days prior to registration
- History or evidence of melanoma associated with immunodeficiency states (e.g., hereditary immune deficiency, organ transplant, or leukemia, requires concomitant treatment with immunosuppressive agents, including CTLA-4 agonists, or chronic oral or systemic steroid medication including physiological replacement doses for adrenal insufficiency
- History of or plan for splenectomy or splenic irradiation
- History or evidence of central nervous system (CNS) metastases
- Active skin lesions (open wounds, severe rash, herpetic lesions, etc.)
- Known history of active tuberculosis
- Known history of Human Immunodeficiency Virus (HIV) (HIV 1/2 antibodies)
- Known acute or chronic hepatitis B or hepatitis C infection (requires negative test)
- Metastatic ocular melanoma patients only: liver radioembolization ≤90 days prior to registration
- No other active second malignancy other than non-melanoma skin cancers and in situ cervical cancers within 3 years of registration. NOTE: A second malignancy is not considered active if all treatment for that malignancy is completed and the patient has been disease-free for at least 3years prior to registration
- No uncontrolled intercurrent illness including, but not limited to:
  - Ongoing or active infection
  - Symptomatic congestive heart failure
  - Unstable angina pectoris
  - Uncontrolled symptomatic cardiac arrhythmia
  - Uncontrolled hypertension (defined as blood pressure >160/90)
- New York Heart Association classification III or IV, known symptomatic coronary artery disease or symptoms of coronary artery disease on systems review, or known cardiac arrhythmias
- Active CNS disorder or seizure disorder or known CNS disease or neurologic symptomatology
- Pregnant or breast-feeding, or planning to become pregnant during study treatment and through 3 months after the last dose of study treatment
- Person of childbearing potential who is unwilling to use two (2) highly effective methods of contraception during study treatment and through 120 days after the last dose of study treatment. (See Appendix III)
- Person able to father a child who is unwilling to use a highly effective method of contraception during study treatment and through 120 days after the last dose of study treatment.

# Supplementary Figures and Tables

## Supplementary Figures


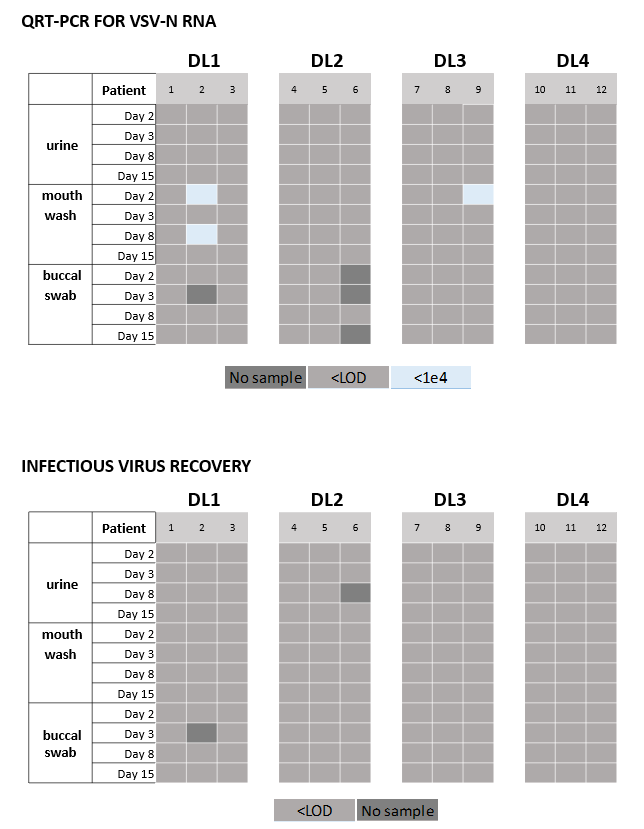


**Supplementary Figure 1.** Viral shedding was measured with quantitative RT-PCR of urine, mouth wash, and buccal swab sample. VSV-N was detected only in the mouth wash at DL1 (Days 2 ND 8) and DL3 (Day 2)
